# Occupational stress and occupational well-being among rural preschool teachers: a moderated mediation model of job burnout and professional identity

**DOI:** 10.3389/fpubh.2026.1954719

**Published:** 2026-09-22

**Authors:** Ruiwei Yu, Keyi Cao, Yingjie Wang, Huilan Yue, Wengui Zhang, Jing Li, Dan Chen

**Affiliations:** 1 College of Education, Huzhou Normal University, Huzhou, Zhejiang, China; 2 Danfeng Kindergarten, Hangzhou, Zhejiang, China

**Keywords:** job burnout, occupational stress, occupational well-being, professional identity, rural preschool teachers

## Abstract

**Introduction:**

The occupational well-being of rural preschool teachers is important for their professional development, mental health, and the quality of preschool education. However, persistent resource constraints in rural education may pose challenges to teachers’ occupational well-being. This study examined the association between occupational stress and occupational well-being among rural preschool teachers, focusing on the indirect role of job burnout and the moderating role of professional identity.

**Methods:**

A cross-sectional questionnaire survey was conducted among 3,228 rural preschool teachers in Zhejiang Province, China, of whom 98.8% were women. Participants completed validated measures of occupational stress, job burnout, professional identity, and occupational well-being. The hypothesized associations were examined using a moderated mediation model.

**Results:**

Occupational stress was significantly and negatively associated with occupational well-being. A significant indirect association between occupational stress and occupational well-being was observed through job burnout, while the direct association remained significant. Professional identity moderated the association between occupational stress and job burnout: this positive association was weaker, but remained statistically significant, at higher levels of professional identity. However, professional identity did not moderate the direct association between occupational stress and occupational well-being.

**Discussion:**

The findings suggest that professional identity may serve as a modest personal resource that attenuates the association between occupational stress and job burnout. They also highlight the importance of reducing job demands and strengthening organizational resources to support the occupational well-being of rural preschool teachers.

## 1 Introduction

In a global context, early childhood education is widely acknowledged as the cornerstone for lifelong learning and comprehensive development (1, 2). This recognition has spurred international efforts to ensure universal access to quality early childhood development, care, and pre-primary education by 2030 (3). Reflecting these global priorities, China has significantly strengthened its policy support for early childhood education (4, 5). These emphasize the enhancement of infrastructure, teacher training, and financial investment to reduce urban–rural disparities. In particular, policies specifically targeting rural early childhood education aim to ensure that children in rural areas have equitable access to quality education (6). Against this policy backdrop, the occupational well-being of rural preschool teachers becomes especially critical, as it directly influences teaching quality, professional sustainability, and overall educational outcomes in these communities. Consequently, investigating the unique factors that affect the occupational well-being of rural preschool teachers is essential for developing a sustainable and high-quality early childhood education system.

Occupational well-being is generally defined as the sustained and positive emotional experience derived from fulfilling one’s needs in the workplace (7). Previous research has demonstrated that the occupational well-being of rural preschool teachers is a critical factor influencing their career development (8, 9). Higher levels of occupational well-being positively influence teachers’ work engagement, job satisfaction, and retention, ultimately enhancing the quality of early childhood education (10, 11). Conversely, lower levels of occupational well-being can negatively impact teachers’ enthusiasm for their work and their ability to create a supportive learning environment for children (12–14). Therefore, improving the occupational well-being of rural preschool teachers is not only essential for stabilizing the teaching workforce but also crucial for improving the quality of preschool education.

In China, the development of preschool education has profound implications for the overall social and economic revitalization of rural areas. Preschool education plays a foundational role in the long-term development of rural students, and the stable supply of rural preschool teachers is a prerequisite for the sustained development of rural preschool education (15). The development of rural preschool education in China is profoundly shaped by the long-standing urban–rural dual structure, which has led to persistent disparities in resource allocation and policy support. These disparities contribute to a range of challenges, including difficulties in attracting students (16, 17), insufficient educational resources, low social recognition, limited staffing quotas, and unstable, inadequate income (18). Such challenges directly impact the occupational well-being of rural preschool teachers, whose job satisfaction and sense of fulfillment are notably lower than those of urban teachers (19). The rising teacher attrition rate has further exacerbated the shortage of qualified staff in rural preschool education (20). Consequently, exploring strategies to improve the occupational well-being of rural preschool teachers has become a critical factor in enhancing the overall quality of rural preschool education.

Among the various factors influencing teachers’ occupational well-being, occupational stress is particularly significant. Occupational stress encompasses four dimensions: work difficulty and challenges, work responsibilities and rewards, work intensity, and management systems and career development (21). Previous research suggests that the impact of occupational stress on occupational well-being can be both positive and negative, though the negative effects are more frequently observed (22, 23). Globally, teaching is considered one of the most high-stress professions (24, 25). In high-pressure environments, teachers are prone to negative emotions and diminished enthusiasm, both of which can adversely affect their occupational well-being (26). Although extensive research has examined the impact of occupational stress on teachers’ occupational well-being, relatively few studies have focused on rural preschool teachers. Compared to other educators, rural preschool teachers face greater responsibilities, higher work intensity, and more demanding professional requirements. Their unique work environments expose them to heightened levels of both physiological and psychological stress (27). Based on these considerations, this study posits the following hypothesis H1: Occupational stress significantly and negatively associated with the occupational well-being of rural preschool teachers.

Although previous studies generally suggest that occupational stress significantly negatively predicts occupational well-being, research indicates that this relationship is not purely direct. Job burnout, another critical influencing factor, plays a mediating role in this connection. Job burnout is defined as a state of exhaustion encompassing behavior, emotions, attitudes, and thoughts, arising when individuals are unable to effectively manage work-related stress (28, 29). Teachers, particularly rural preschool teachers, are highly vulnerable to job burnout (30). Studies have revealed that job burnout among rural preschool teachers is notably severe, often leading to adverse effects on their physical and psychological health (31, 32).

Research indicates that job burnout mediates the relationship between occupational stress and occupational well-being (33, 34). According to the health-impairment process of the JD-R model, sustained job demands are associated with the depletion of employees’ physical and psychological resources, which may be manifested in job burnout and lower occupational well-being (35, 36). Occupational stress is a critical factor contributing to job burnout, with research showing that it significantly and positively predicts teacher burnout (37–39). Moreover, studies have consistently demonstrated that job burnout negatively predicts occupational well-being, indicating that higher levels of burnout correspond to lower levels of occupational well-being (40–42). With the ongoing advancement of urbanization, rural preschool teachers face unique identity and psychological challenges (43). These challenges, coupled with significant pressures from students, parents, and career development demands, often result in heightened job burnout and diminished occupational well-being. Consequently, this study hypothesizes H2: Job burnout statistically mediates the relationship between occupational stress and occupational well-being among rural preschool teachers.

While occupational stress may influence occupational well-being through job burnout, research demonstrates that teachers in similar environments often exhibit differing work conditions (44, 45). This suggests that certain factors may provide moderating support. According to the Job Demands-Resources (JD-R) model, positive work resources can mitigate the adverse effects of work demands on occupational well-being (46). These resources can be categorized into internal individual resources and external work resources (47). Extensive research has identified professional identity as a critical internal resource. Professional identity encompasses teachers’ positive cognitions, experiences, and behavioral tendencies toward their profession and serves as an essential individual resource (48, 49). A strong professional identity functions as a positive emotional resource, buffering the psychological strain caused by excessive occupational stress and work demands (50, 51). Consequently, professional identity may alleviate the negative effects of occupational stress. Based on this, the study hypothesizes H3: Professional identity moderates the association (a) between occupational stress and job burnout and (b) between occupational stress and occupational well-being among rural preschool teachers.

In summary, this study seeks to investigate the association between occupational stress and occupational well-being among rural preschool teachers using a moderated mediation model (see Figure 1). Specifically, it explored the mediating role of job burnout and the moderating effect of professional identity. The study aims to provide valuable research insights for alleviating the decline in occupational well-being among rural preschool teachers.

**Figure 1:**
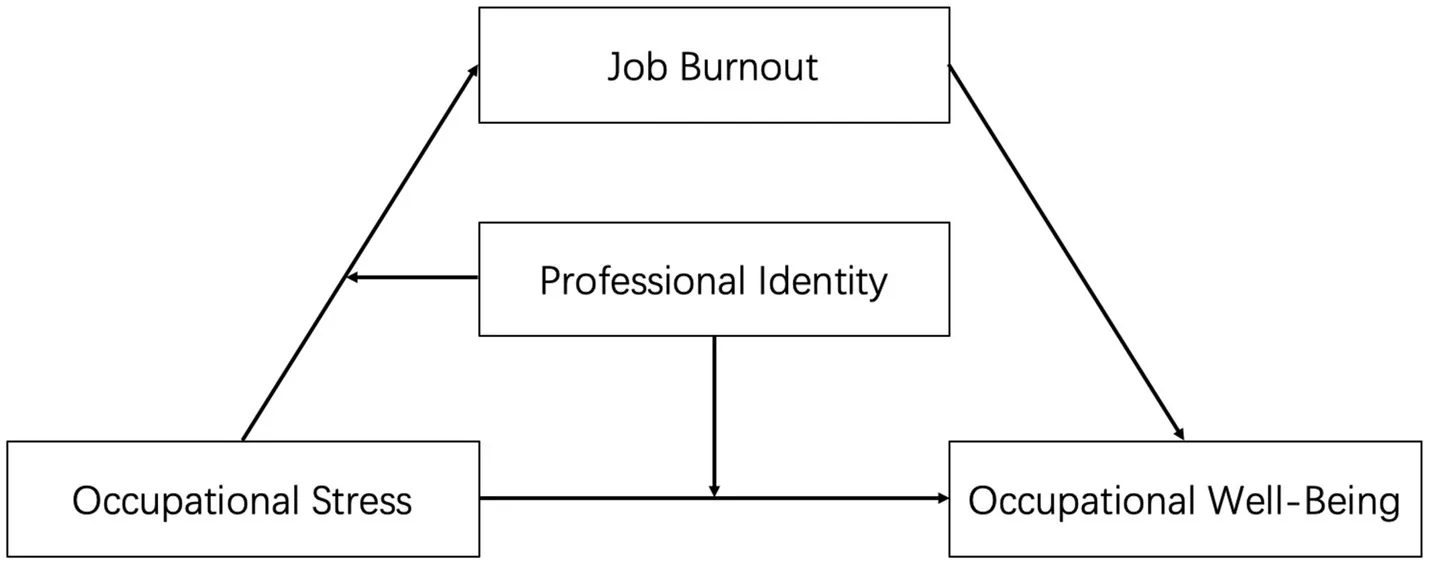
Hypothetical model of a moderated mediation effect.

## 2 Methods

### 2.1 Research participants

This cross-sectional questionnaire survey was conducted in November 2023 among preschool teachers from 160 rural kindergartens in Zhejiang Province, China. Local early childhood education administrators assisted with questionnaire distribution. Each participating kindergarten had 9–16 classes, and two classroom teachers from each class were invited to participate. Thus, 18–32 teachers were invited from each kindergarten, for a total of 3,440 teachers, with an average of 21.5 teachers per kindergarten. Of the 3,440 teachers invited, 3,366 returned questionnaires, yielding a response rate of 97.8%. Following data screening, 138 questionnaires were excluded because of missing data or straight-line response patterns (i.e., selecting the same response option for all scale items). The final analytic sample consisted of 3,228 teachers, representing 93.8% of those invited.

Among the respondents, 98.8% were female (3,189 participants), and 1.2% were male (39 participants). The teachers’ ages ranged from 18 to 64 years, with an average age of 31.8 years (*SD* = 7.1). In terms of teaching experience, the majority of participants had less than 10 years of experience: 35.9% had less than 5 years, 28.2% had 6 to 10 years, 18.3% had 11 to 15 years, 9.7% had 16 to 20 years, and 7.9% had more than 20 years of experience. Regarding educational background, most teachers held a bachelor’s degree (65.5%), while others possessed an associate degree (33.2%) or a high school diploma (1.2%).

### 2.2 Research instruments

#### 2.2.1 Occupational well-being

The Occupational well-being Scale for Preschool Teachers, developed by Wang (2013), was used to measure occupational well-being (7). The scale includes four dimensions: psychological well-being, emotional well-being, social well-being, and cognitive well-being, with a total of 15 items. A 3-point Likert scale was employed, with responses ranging from “completely disagree” to “completely agree,” scored from 1 to 3. Higher scores indicate stronger occupational well-being. In this study, the scale demonstrated excellent reliability, with a Cronbach’s α coefficient of 0.96.

#### 2.2.2 Professional identity

The Professional Identity Scale, developed by Wei and colleagues in the context of China (48), was employed to measure professional identity. This scale consists of four dimensions: role value, professional behavioral tendencies, professional values, and a sense of professional belonging, with a total of 18 items. Responses were recorded on a 5-point Likert scale ranging from “strongly disagree” to “strongly agree,” scored from 1 to 5. Higher scores indicate stronger professional identity. In this study, the scale’s Cronbach’s α coefficient was 0.95.

#### 2.2.3 Occupational stress

The Occupational Stress Questionnaire for Preschool Teachers, developed by Alatambagan and Liu (21), was used to measure occupational stress. This scale includes four dimensions: work difficulty and challenges, work responsibilities and rewards, work intensity, and management systems and career development, with a total of 18 items. Responses were rated on a 5-point Likert scale, ranging from “no stress” to “extreme stress,” scored from 1 to 5. Higher scores indicate greater perceived occupational stress. In this study, the scale’s Cronbach’s α coefficient was 0.96.

#### 2.2.4 Job burnout

The Maslach Burnout Inventory–Educators Survey (MBI-ES), developed by Maslach, Jackson, and Schwab (1986) (52), and translated into Chinese by Chan and Hui (53), was used to measure job burnout. The scale includes three dimensions: emotional exhaustion, depersonalization, and reduced personal accomplishment, with a total of 22 items. Responses were recorded on a 7-point Likert scale ranging from “never” to “daily,” scored from 1 to 7. Because higher scores on the reduced personal accomplishment items indicate greater personal accomplishment and thus lower burnout, these items were reverse-scored using the formula 8 − the original score. For the present analyses, an overall burnout score was calculated as the mean of all 22 items after reverse-scoring the personal accomplishment items. Higher scores indicate higher levels of job burnout. In this study, the scale’s Cronbach’s α coefficient was 0.91.

### 2.3 Research procedure

This study actively collaborated with preschool education administrators in 21 rural regions of Zhejiang Province. With their support, random samples of preschools were selected in each region. Effective communication was established with preschool principals, and with their cooperation, online survey links were distributed to all teachers. To ensure compliance and protect participants’ rights, an informed consent form was embedded on the first page of the online survey. All participants were informed about the purpose, content, and details of the study before completing the survey, and consent was obtained prior to participation.

### 2.4 Ethical approval and informed consent

This study was reviewed and approved by the academic research office of the College of Education, Huzhou University. All procedures involving human participants adhered to the principles outlined in the Declaration of Helsinki. Informed consent was obtained from all participants prior to their inclusion in the study.

### 2.5 Data analysis

The collected data were analyzed using SPSS 26.0 for descriptive statistics, reliability analysis, and correlation analysis. Additionally, moderated mediation analysis was conducted using Model 8 of the SPSS PROCESS macro (54). Because gender showed very limited variation in this predominantly female sample (98.8%), it was not included as a covariate. Age, teaching experience, educational background, and income were controlled in all regression models.

### 2.6 Common method bias test

To examine common method bias, an exploratory factor analysis (EFA) was performed on the valid responses. The results showed nine factors with eigenvalues greater than 1, with the first factor explaining 17.57% of the variance, which is well below the 40% threshold. Thus, Harman’s single-factor test did not indicate that common method bias was a serious concern in the present study. Nevertheless, this test provides only a preliminary assessment and cannot rule out common method bias entirely.

## 3 Results

### 3.1 Preliminary analysis

The means, standard deviations, and correlations of key variables, including occupational stress, job burnout, professional identity, and occupational well-being, are presented in Table 1. The results indicate significant relationships among the primary variables. Specifically, occupational stress was significantly negatively correlated with professional identity and occupational well-being, but significantly positively correlated with job burnout. Professional identity was significantly positively correlated with occupational well-being and significantly negatively correlated with job burnout. Additionally, job burnout was significantly negatively correlated with occupational well-being. These findings suggest the necessity of further examining the relationships among these variables.

**Table 1:** Descriptive statistics and correlation analysis of variables.

Variables	1	2	3	4	5	6	7	8
1. Age	—							
2. Teaching experience	0.84^***^	—						
3. Educational background	0.08^***^	0.12^***^	—					
4. Income	0.08^***^	0.15^***^	0.33^***^	—				
5. Occupational stress	−0.02	0.01	0.02	−0.02	—			
6. Occupational well-being	0.12^***^	0.09^***^	0.01	0.04^*^	−0.51^***^	—		
7. Professional Identity	0.09^***^	0.08^***^	0.06^***^	0.07^***^	−0.28^***^	0.43^***^	—	
8. Job burnout	−0.12^***^	−0.08^***^	−0.01	−0.00	0.60^***^	−0.59^***^	−0.48^***^	—
*M*	31.77	9.42			2.49	2.31	4.26	3.09
*SD*	7.08	7.15			0.91	0.47	0.63	0.90

^*^p < 0.05, ^***^p < 0.001; education background: 1 = high school, 2 = junior college, and 3 = bachelor’s degree; income: 1 = under 80,000 RMB per year, 2 = 80,000–100,000 RMB per year, 3 = 100,000–120,000 RMB per year, and 4 = above 120,000 RMB per year.

Regarding control variables, demographic factors (age, years of teaching experience, education level, and income) showed varying degrees of correlation with occupational stress, job burnout, professional identity, and occupational well-being. Therefore, these demographic variables were included as control variables in subsequent models.

### 3.2 The association between occupational stress and occupational well-being

To estimate the overall association between occupational stress and occupational well-being, a multiple linear regression analysis was conducted. Occupational well-being was entered as the dependent variable, occupational stress as the independent variable, and age, teaching experience, educational background, and income as covariates. The results indicated that overall regression model was statistically significant, *R* = 0.52, *R^2^* = 0.27, *F*_(5,3,222)_ = 241.25, *p* < 0.001, explaining 27% of the variance in occupational well-being. After controlling for the demographic covariates, occupational stress was significantly and negatively associated with occupational well-being (*β* = −0.51, *SE* = 0.02, *t* = −33.77, *p* < 0.001). Thus, higher occupational stress was associated with lower occupational well-being, providing support for H1. Among the covariates, age was positively associated with occupational well-being (*β* = 0.09, *SE* = 0.004, *t* = 3.37, *p* < 0.001), whereas teaching experience, educational background, and income were not significantly associated with occupational well-being.

### 3.3 Moderated mediation analysis

A conditional process analysis was conducted using Model 8 in the SPSS PROCESS macro to examine whether the association between occupational stress and occupational well-being was statistically mediated by job burnout and whether professional identity moderated the relevant associations. Demographic factors (age, teaching experience, education level, and income) were included as control variables due to their significant correlations with the main study variables. All variables were standardized prior to analysis.

The results (see Table 2 and Figure 2) revealed that, after job burnout and professional identity was included in the model, occupational stress remained significantly and negatively associated with occupational well-being (*β* = −0.25, *p* < 0.001). Occupational stress was also significantly and positively associated with job burnout (*β* = 0.50, *p* < 0.001), whereas job burnout was significantly and negatively associated with occupational well-being (*β* = −0.35, *p* < 0.001). Using bias-corrected bootstrapping with 5,000 iterations, the indirect effect was estimated to be −0.26, with a 95% confidence interval (CI) of [−0.29, −0.23], indicating a significant mediation effect.

**Table 2:** Moderation of the association between occupational stress and job burnout by professional identity.

Dependent variables	Independent variables	*R*	*R^2^*	*F*	*B*	*β*	*SE*	*t*	*p*	95% CI
Job burnout	Age	0.69	0.47	405.87^***^	−0.02	−0.02	0.003	−5.13^***^	< 0.001	[−0.02,-0.01]
	Teaching experience				0.01	0.01	0.003	1.92	0.055	[0.000,0.01]
	Educational background				−0.02	−0.02	0.03	−0.81	0.417	[−0.07,0.03]
	Income				0.04	0.04	0.02	2.79^**^	0.005	[0.01,0.07]
	Occupational stress				0.76	0.50	0.01	37.56^***^	< 0.001	[0.48,0.53]
	Professional identity				−0.33	−0.33	0.01	−24.56^***^	< 0.001	[−0.36,-0.31]
	Occupational stress × Professional identity				−0.06	−0.04	0.01	−3.13^**^	0.002	[−0.06,−0.01]
Occupational well-being	Age	0.65	0.42	293.88^***^	0.002	0.003	0.004	0.964	0.335	[−0.004,0.01]
	Teaching experience				0.002	0.005	0.004	1.313	0.189	[−0.002,0.01]
	Educational background				-0.01	−0.02	0.028	−0.613	0.540	[−0.07,0.04]
	Income				0.01	0.02	0.016	1.025	0.305	[−0.02,0.05]
	Occupational stress				−0.15	−0.25	0.017	−14.916^***^	< 0.001	[−0.28,-0.22]
	Job burnout				−0.18	−0.35	0.018	−18.76^***^	< 0.001	[−0.38,-0.31]
	Professional identity				0.13	0.19	0.015	12.692^***^	< 0.001	[0.16,0.23]
	Occupational stress × Professional identity				0.01	0.01	0.013	0.451	0.663	[−0.02,0.03]

^**^p < 0.01; ^***^p < 0.001.; B = unstandardized regression coefficient; β = standardized regression coefficient.

**Figure 2:**
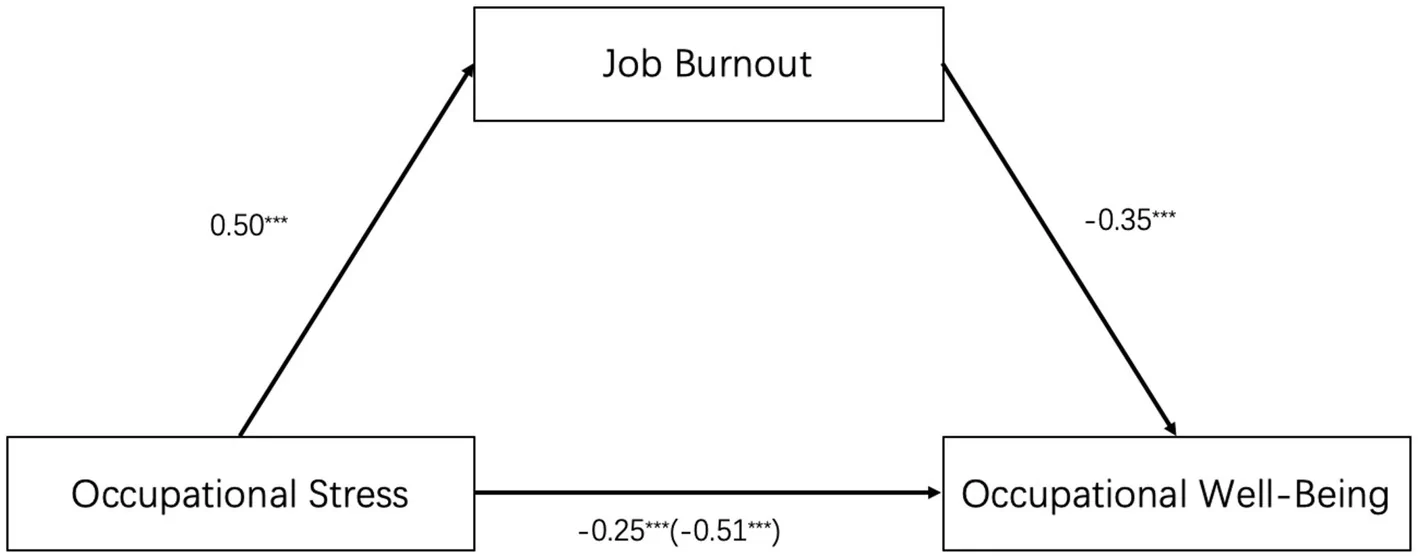
The indirect association between occupational stress and occupational well-being through job burnout. The coefficient outside parentheses represents the direct association after job burnout was included, whereas the coefficient in parentheses represents the total association before job burnout was included. ^***^p < 0.001.

The results also revealed that professional identity did not significantly moderate the relationship between occupational stress and occupational well-being (*β* = 0.01, *t* = 0.45, *p* > 0.05, 95% CI [−0.02, 0.03]). However, professional identity significantly moderated the relationship between occupational stress and job burnout (*β* = −0.04, *t* = −3.13, *p* < 0.01, 95% CI [−0.06, −0.01]). This indicates that professional identity plays a significant moderating role in the relationship between occupational stress and job burnout. The indirect effects of occupational stress on Occupational well-being via job burnout at high level of professional identity (*β* = −0.16, *SE* = 0.013, 95% CI [−0.19, −0.14]) and low level of professional identity (*β* = −0.19, *SE* = 0.013, 95% CI [−0.21, −0.16]). Moreover, the index of moderated mediation was statistically significant (*Index* = 0.01, *BootSE* = 0.005, 95% CI [0.003, 0.023]).

In order to reveal the essence of the interaction, we conducted the simple slopes at lower (−1SD) and higher (+1SD) levels of professional identity (Figure 3). The analysis revealed that in the high level of professional identity, occupational stress was significantly and positively associated with job burnout (*β* = 0.47, *t* = 26.12, *p* < 0.001, 95% CI [0.43, 0.50]). A similar positive association was observed at lower levels of professional identity (*β* = 0.54, *t* = 29.41, *p* < 0.001, 95% CI [0.51, 0.58]). However, the positive association between occupational stress and job burnout was weaker at higher levels of professional identity.

**Figure 3:**
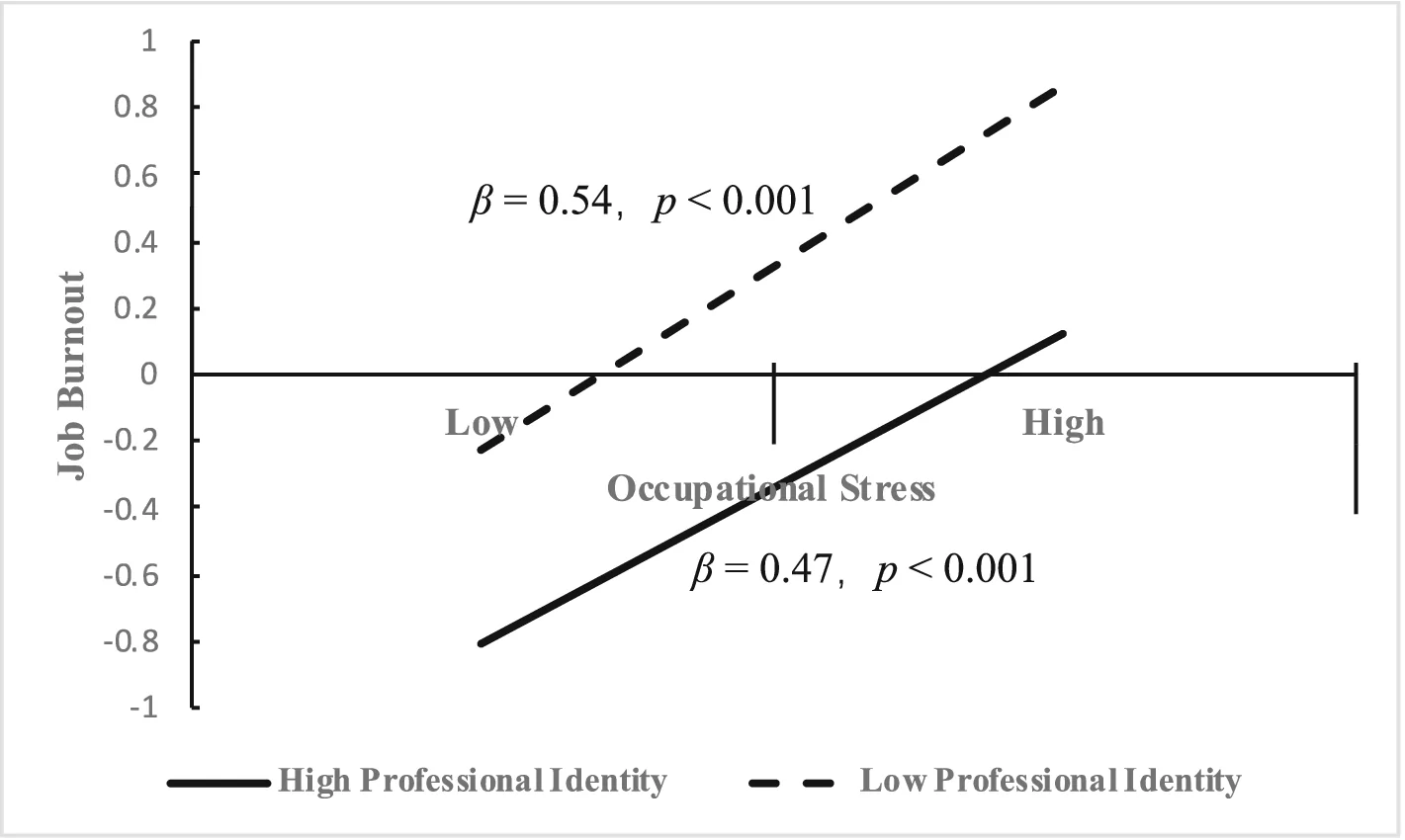
The moderating role of professional identity in the association between occupational stress and job burnout. Low and high levels of professional identity represent one standard deviation below and above the mean, respectively.

Further simple slope analysis showed that the positive association between occupational stress and job burnout was stronger among rural preschool teachers with lower professional identity. In contrast, this association was weaker among teachers with higher professional identity.

## 4 Discussion

### 4.1 Relationship between occupational stress and occupational well-being

The present study found that occupational stress was negatively associated with occupational well-being in a predominantly female sample of rural preschool teachers, consistent with previous research (55) and supporting H1. Rural preschool teaching combines caregiving and educational responsibilities and requires teachers to respond to children’s developmental needs, classroom management, and parental expectations. These overlapping responsibilities make occupational stress a particular concern for this group (27, 56). Rural preschool teachers also face fragmented work, limited opportunities for professional development, and difficulties balancing work and family responsibilities (27, 33, 57, 58). At the same time, their financial compensation and social recognition may not be commensurate with their work demands (15, 58, 59). These difficulties are compounded by urban–rural disparities in educational resources and professional-development opportunities (17).

The JD–R model offers a useful explanation for this association. Heavy teaching and administrative responsibilities constitute substantial job demands, whereas inadequate compensation, limited career-development opportunities, and insufficient social recognition reflect a lack of job resources (58, 59). A recent study of rural preschool teachers in China similarly found that effort–reward imbalance was associated with lower occupational well-being (59). Although effort–reward imbalance and occupational stress are distinct constructs, both findings point to the adverse work-related experiences associated with high demands and inadequate resources. The present results therefore underscore the need to reduce unnecessary workloads and provide rural preschool teachers with adequate resources, professional-development opportunities, and occupational recognition.

### 4.2 The mediating role of burnout

The results revealed a significant indirect association between occupational stress and occupational well-being through job burnout, supporting H2 and extending previous findings (60, 61). A comparable pattern was reported in a recent study among rural preschool teachers in China, in which emotional exhaustion statistically mediated the association between effort–reward imbalance and occupational well-being (59). Emotional exhaustion is a central component of job burnout, and this evidence provides relevant support for the role of burnout in adverse occupational experiences among rural preschool teachers.

This finding is consistent with the health impairment process of the JD–R model. Sustained job demands may consume teachers’ physical and psychological resources, leaving them more vulnerable to burnout and poorer occupational well-being (35). In China, expectations concerning teachers’ professional conduct, moral responsibility, and commitment may add to their perceived occupational obligations (62, 63). Research on early childhood teachers and rural teachers has likewise emphasized the commitment and adaptability required to remain in demanding and resource-constrained settings (64, 65). Within the JD–R framework, these cultural and occupational expectations can be understood as contextual demands that add to teachers’ existing workloads.

For rural preschool teachers, sustained workloads, parental expectations, inadequate organizational support, and limited educational resources may be associated with emotional exhaustion, depersonalization, and reduced personal accomplishment (27, 66). These experiences can undermine teachers’ motivation and their evaluations of work, thereby contributing to lower occupational well-being (60, 61).

Job burnout, however, did not account for the entire association between occupational stress and occupational well-being. The direct association remained significant after job burnout was included in the model, indicating that other processes may also be involved. Occupational well-being encompasses a wide range of work-related experiences. Its association with occupational stress may also involve work–family conflict (33), dissatisfaction with occupational rewards (59), limited organizational support and decision-making opportunities (58), and restricted career development and social recognition (15, 27, 58). Psychological responses such as lower job satisfaction, diminished psychological empowerment, and less adaptive coping may offer additional explanations, although these possibilities were not examined in the present study.

Because the data were cross-sectional, the indirect effect does not establish the temporal or causal ordering of occupational stress, job burnout, and occupational well-being. Reciprocal associations are also possible. Longitudinal research is needed to determine whether occupational stress precedes burnout and subsequent changes in occupational well-being.

### 4.3 The moderating role of professional identity

Professional identity moderated the association between occupational stress and job burnout, supporting H3a and the resource-buffering proposition of the JD–R model (47, 67). Personal resources within the JD–R framework are psychological characteristics that help individuals manage their work environment and respond to job demands (47). Professional identity can be regarded as an occupation-specific personal resource because it reflects teachers’ understanding of, emotional attachment to, and commitment to their professional roles (48, 49). It may provide a sense of professional meaning and role coherence that helps teachers interpret occupational demands and sustain coping under stress.

Evidence from rural preschool teachers also suggests that personal resources are relevant to the way teachers respond to unfavorable working conditions. Psychological capital, for example, has been found to moderate associations between effort–reward imbalance, emotional exhaustion, and occupational well-being (59). Although psychological capital differs from professional identity, this finding supports the broader proposition that personal resources may shape teachers’ responses to demanding work environments.

Professional identity may be particularly important in rural preschool settings, where teachers often work with limited educational resources and experience demanding working conditions and work–family conflict (68, 69). Stronger professional identity has been associated with greater career adaptability (51), lower job burnout, and more favorable work-related psychological states (56, 70, 71). For rural teachers, it may also reflect a commitment to rural education and to the development of the children they teach (72).

The simple slope analysis showed that the positive association between occupational stress and job burnout was weaker among teachers with higher professional identity. The association nevertheless remained significant at all examined levels of professional identity, indicating attenuation rather than elimination of the effect. Moreover, the interaction accounted for only a small increase in explained variance (ΔR^2^ = 0.002). The buffering role of professional identity should therefore be interpreted as statistically significant but modest in magnitude.

Professional identity did not moderate the direct association between occupational stress and occupational well-being, and H3b was therefore not supported. One explanation concerns the relative proximity of the constructs. Burnout is a proximal response to sustained job demands, whereas professional identity directly concerns how teachers understand their work and professional roles. Professional identity may therefore be more relevant to teachers’ immediate burnout responses than to their overall occupational well-being.

Occupational well-being is broader and is shaped by working conditions, interpersonal relationships, organizational support, and perceptions of personal accomplishment (73). Professional identity may help teachers retain meaning and commitment in their work, but it cannot compensate for inadequate staffing, low salaries, insufficient teaching resources, limited career opportunities, or work–family conflict (15, 33, 58, 59, 69, 70). These structural conditions may continue to affect teachers’ occupational well-being regardless of their level of professional identity.

The nonsignificant interaction does not imply that professional identity is unrelated to occupational well-being. Professional identity was positively associated with occupational well-being in the outcome model, suggesting an additive rather than an interactive association. Teachers with stronger professional identity tended to report higher occupational well-being, but professional identity did not alter the strength of the direct association between occupational stress and occupational well-being.

Overall, H3 was partially supported: H3a was supported, whereas H3b was not. The moderated mediation results indicate that professional identity conditioned the indirect association between occupational stress and occupational well-being through its moderation of the occupational stress–burnout path. Professional identity may therefore serve as a modest personal resource in relation to burnout, but it cannot replace the organizational and structural resources needed to improve rural preschool teachers’ occupational well-being.

## 5 Limitations and future research directions

Although this study provides valuable insights into the associations among occupational stress, burnout, professional identity, and the occupational well-being of rural preschool teachers, several limitations should be addressed in future research.

First, all participants were rural preschool teachers from Zhejiang Province, which limits the generalizability of the findings to teachers in other regions with different economic, social, and educational conditions. In addition, the cross-sectional design does not establish the temporal or causal ordering of occupational stress, job burnout, professional identity, and occupational well-being. Although the analysis identified a significant indirect association through job burnout, it cannot determine whether occupational stress preceded burnout and subsequent differences in occupational well-being. Reverse or reciprocal associations are also possible. Future research should include rural preschool teachers from a wider range of regions and use multiwave longitudinal designs to examine these relationships across time.

Second, female participants accounted for 98.8% of the sample. This marked imbalance may reflect the female-dominated composition of the preschool teaching workforce, but the findings primarily characterize the occupational experiences of female rural preschool teachers. The small number of male participants did not allow meaningful gender comparisons, and the findings should not be assumed to apply equally to male teachers. Future studies could recruit more male rural preschool teachers to examine whether the observed associations vary by gender.

Third, teachers were recruited from 160 rural kindergartens, and those working in the same kindergarten may have shared some aspects of their organizational and working environments. Because kindergarten identifiers were not retained in the analytic dataset, the extent of any within-kindergarten clustering could not be directly evaluated. Future studies should retain kindergarten identifiers and consider multilevel models or cluster-robust analyses where appropriate.

Fourth, job burnout was analyzed as an overall construct. The present study therefore cannot determine whether emotional exhaustion, depersonalization, and reduced personal accomplishment contribute differently to the indirect association between occupational stress and occupational well-being. Examining these dimensions separately, or as parallel mediators, may provide a more detailed account of how different aspects of burnout are associated with teachers’ occupational well-being.

Finally, all variables were measured using self-report questionnaires collected at a single time point. Although the common method bias test did not indicate a serious problem, shared method variance and socially desirable responding cannot be ruled out. The model also did not include other factors that may be relevant to occupational well-being, such as social support, work–family conflict, organizational climate, working conditions, and individual characteristics. Future studies could combine teacher reports with data from other sources and examine additional psychological and organizational mechanisms.

## 6 Conclusion and recommendations

### 6.1 Conclusion

In this predominantly female sample of 3,228 rural preschool teachers in Zhejiang Province, three findings emerged. First, Occupational stress was significantly and negatively associated with occupational well-being. Second, Job burnout partially mediates the association between occupational stress and occupational well-being. Third, the association between occupational stress and job burnout was weaker among teachers with stronger professional identity. Professional identity did not, however, moderate the direct association between occupational stress and occupational well-being. Professional identity may therefore have a modest buffering role in the stress–burnout relationship.

### 6.2 Recommendations

Based on the study’s findings, the following recommendations are made:

Government and educational administration should pay attention to the issue of occupational stress among rural preschool teachers and take effective measures to reduce their workload, such as improving working conditions, increasing salaries, and providing more professional development opportunities.

Schools should strengthen the development of teachers’ professional identity by organizing training, providing psychological support, and helping teachers establish correct professional values and a sense of mission, thus enhancing their ability to cope with stress.

Teachers should focus on preventing and managing burnout by taking regular breaks, establishing a healthy work-life balance, and participating in professional development activities to reduce burnout and enhance occupational well-being.

## Data Availability

The raw data supporting the conclusions of this article will be made available by the authors, without undue reservation.
